# Predictive modeling of treatment-related thyroid dysfunction and prognostic implication in advanced nasopharyngeal carcinoma with PD-1 inhibitors

**DOI:** 10.1093/oncolo/oyag066

**Published:** 2026-02-25

**Authors:** Yubin Hu, Xinyi Hong, Yi Li, Honghong Zhang, Mohammad Abidullah Warsi, Ronghui Chen, Chaoying Lin, Peidong Ou, Cuibo Lin, Sufang Qiu

**Affiliations:** Department of Radiation Oncology, Clinical Oncology School of Fujian Medical University, Fujian Cancer Hospital (Fujian Branch of Fudan University Shanghai Cancer Center), Fujian 350014, People's Republic of China; Department of Radiation Oncology, Clinical Oncology School of Fujian Medical University, Fujian Cancer Hospital (Fujian Branch of Fudan University Shanghai Cancer Center), Fujian 350014, People's Republic of China; Department of Radiation Oncology, Clinical Oncology School of Fujian Medical University, Fujian Cancer Hospital (Fujian Branch of Fudan University Shanghai Cancer Center), Fujian 350014, People's Republic of China; Department of Radiation Oncology, Xiang’an Hospital of Xiamen University, School of Medicine, Xiamen University, Xiamen 361005, People's Republic of China; Department of Radiation Oncology, Clinical Oncology School of Fujian Medical University, Fujian Cancer Hospital (Fujian Branch of Fudan University Shanghai Cancer Center), Fujian 350014, People's Republic of China; Department of Radiation Oncology, Clinical Oncology School of Fujian Medical University, Fujian Cancer Hospital (Fujian Branch of Fudan University Shanghai Cancer Center), Fujian 350014, People's Republic of China; Department of Radiation Oncology, Clinical Oncology School of Fujian Medical University, Fujian Cancer Hospital (Fujian Branch of Fudan University Shanghai Cancer Center), Fujian 350014, People's Republic of China; Department of Radiation Oncology, Clinical Oncology School of Fujian Medical University, Fujian Cancer Hospital (Fujian Branch of Fudan University Shanghai Cancer Center), Fujian 350014, People's Republic of China; Department of Radiation Oncology, Clinical Oncology School of Fujian Medical University, Fujian Cancer Hospital (Fujian Branch of Fudan University Shanghai Cancer Center), Fujian 350014, People's Republic of China; Department of Radiation Oncology, Clinical Oncology School of Fujian Medical University, Fujian Cancer Hospital (Fujian Branch of Fudan University Shanghai Cancer Center), Fujian 350014, People's Republic of China; Fujian Key Laboratory of Translational Cancer Medicine, Fujian 350014, People's Republic of China

**Keywords:** nasopharyngeal carcinoma, immune checkpoint inhibitors, thyroid dysfunction

## Abstract

**Background:**

Treatment-related thyroid dysfunction (trTD) frequently occurs in advanced nasopharyngeal carcinoma (NPC) patients treated with PD-1 inhibitors. Its clinical features, incidence, and prognostic value remain unclear. This study aimed to characterize trTD and assess its impact on survival.

**Methods:**

We retrospectively analyzed 168 advanced NPC patients who received PD-1 inhibitor therapy between 2019 and 2022. Cox proportional hazards models and Kaplan–Meier analysis were used to assess the prognostic significance of trTD. Clinical factors associated with trTD were identified through logistic regression. A predictive nomogram was constructed and evaluated using receiver operating characteristic (ROC) and calibration curves. Spearman correlation analysis was performed to assess the dynamic relationship between thyroid function indicators and treatment duration.

**Results:**

TrTD developed in 49.4% of patients, mostly mild, including hyperthyroidism, hypothyroidism, and biphasic dysfunction. TrTD was independently associated with improved progression free survival (PFS). Female, elevated ALB, ALT, and TBIL were independent risk factors for trTD. The nomogram constructed by combining these factors and thyroid indicators had good prediction accuracy (AUC = 0.781). Dynamic analysis revealed that TSH levels significantly increased after the fourth treatment cycle, preceding changes in FT3 and FT4, suggesting TSH as a potential early biomarker for trTD onset.

**Conclusion:**

This study identifies trTD as a common event associated with improved survival in advanced NPC patients treated with PD-1 inhibitors. A predictive nomogram and early TSH elevation provide valuable tools for risk stratification and personalized immunotherapy monitoring.

## Graphical Abstract

**Graphical Abstract:**
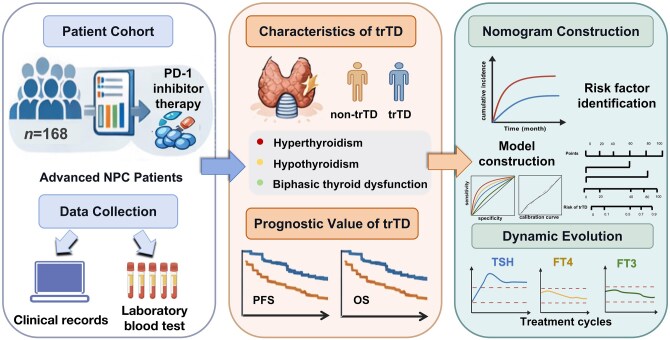

Implications for Practice In patients with advanced NPC receiving PD-1 inhibitors, trTD is common and associated with favorable survival outcomes. A newly developed nomogram incorporating clinical and laboratory parameters offers a practical tool for early risk stratification of trTD. In clinical practice, high-risk patients particularly after ≥5 cycles of PD-1 therapy, should undergo more frequent thyroid monitoring and early endocrine consultation, while low-risk patients may have intervals safely extended. This approach enables early detection, prevents treatment interruptions, and optimizes immunotherapy outcomes. Furthermore, the prognostic value of trTD can inform treatment decisions and guide individualized immunotherapy strategies.

## Introduction

Nasopharyngeal carcinoma (NPC) is a highly aggressive malignant tumor with distinct epidemiological and geographic distribution patterns, with high-incidence regions primarily concentrated in southern China and Southeast Asia, over 70% of NPC patients present with locally advanced or metastatic disease at the time of initial diagnosis.^1^ Due to the propensity of NPC for local invasion and hematogenous metastasis, approximately 15%-30% of patients experience distant metastasis or local recurrence at diagnosis or after treatment, which remains the primary cause of treatment failure.^2^^,^^3^ Therefore, there is an urgent need to optimize therapeutic strategies and enhance early risk stratification in NPC.

In recent years, breakthroughs in immunotherapy have provided new hope for patients with advanced NPC. Several large phase II and III clinical trials, including JUPITER-02, POLARIS-02, and CAPTAIN-1st, have demonstrated that programmed cell death-1 (PD-1) inhibitors significantly improve progression-free survival (PFS) and overall survival (OS) in patients with advanced NPC.^4–6^ Based on these findings, the 2025 National Comprehensive Cancer Network (NCCN) guidelines have designated toripalimab as a first-line monotherapy for recurrent or metastatic NPC.^7^ The phase III CONTINUUM study, led by Academician Ma Jun, confirmed that adding sintilimab to standard therapy significantly improved outcomes in locally advanced head and neck cancers, including NPC, reducing the risk of recurrence, metastasis, or death by 41% and markedly increasing 3-year event free survival, distant metastasis free survival, and recurrence free survival rates.^8^ With the increasing adoption of immunotherapy, PD-1 inhibitors in combination with chemoradiotherapy have significantly improved the prognosis of patients with recurrent or metastatic nasopharyngeal carcinoma.

PD-1 inhibitors, a widely used class of immune checkpoint inhibitors (ICIs), exert antitumor activity by enhancing T cell-mediated immune responses but may also induce immune dysregulation affecting multiple organ systems, including the endocrine axis. Among endocrine immune-related adverse events (irAEs), immune-related thyroid dysfunction (irTD) represents the most frequent manifestation.^9^^,^^10^ IrTD typically follow a biphasic pattern, characterized by transient hyperthyroidism followed by hypothyroidism, and is often associated with thyroid autoantibodies, reflecting underlying immune-mediated injury.^11^ Thyroid dysfunction presents with nonspecific symptoms such as fatigue and cold intolerance, and if untreated, may cause cardiovascular complications, interrupt immunotherapy, and worsen long-term outcomes.^12^ Accumulating evidence over recent years has suggested that the development of irTD may serve as a surrogate marker of immune activation and treatment efficacy, and has been consistently associated with favorable clinical outcomes in several solid tumors, including non-small cell lung cancer, renal cell carcinoma, and metastatic melanoma.^11^^,^^13–16^ Anti-PD-1 therapy has increasingly been incorporated into the treatment paradigm for advanced NPC. Early clinical studies in patients with recurrent or metastatic NPC have reported a highly variable incidence of thyroid dysfunction during immunotherapy, ranging from 6.7% to 32%.^17–19^ In addition to immunotherapy, radiotherapy remains the cornerstone of curative treatment for NPC and represents a well-established cause of thyroid injury. High-dose irradiation of the cervical lymphatic drainage regions inevitably exposes the thyroid gland to substantial radiation doses, leading to radiation-induced hypothyroidism (RIHT), which is typically dose-dependent, delayed in onset, and predominantly manifests as permanent hypothyroidism. Previous studies have reported that the incidence of post-radiotherapy thyroid dysfunction in NPC patients ranges from 30% to 50%.^20^^,^^21^ Given that both radiotherapy and ICIs can independently impair thyroid function, their combination in the management of advanced NPC may exert additive or synergistic effects on thyroid injury. Moreover, emerging evidence suggests that radiotherapy may potentiate the immunostimulatory effects of ICIs, thereby amplifying systemic immune activation.^22^ Despite these findings, in the era of immunotherapy, the clinical characteristics, incidence patterns, risk factors, and prognostic significance of trTD in patients with advanced NPC remain insufficiently investigated.

Therefore, this study aims to systematically evaluate the clinical features, incidence, and prognostic impact of trTD in patients with advanced NPC treated with PD-1 inhibitors. In addition, we sought to develop a nomogram for predicting the occurrence of trTD, with the ultimate goal of improving risk stratification and optimizing individualized clinical management strategies.

## Methods

### Patients

A total of patients with advanced NPC diagnosed between January 1, 2019, and December 31, 2022, were identified through the electronic medical records system of Fujian Provincial Cancer Hospital. The following inclusion criteria were applied: (1) histopathological confirmation of NPC according to World Health Organization (WHO) standards, (2) stage III-IV NPC, (3) an Eastern Cooperative Oncology Group (ECOG) performance status score of ≤1, (4) receipt of at least two cycles of PD-1 therapy, (5) availability of complete clinical and laboratory data.

Exclusion criteria were as follows: (1) abnormal thyroid function prior to immunotherapy; (2) history of thyroid disease; (3) presence of other malignancies; (4) severe comorbidities (eg, hepatic/renal dysfunction, infections, psychiatric or cognitive disorders, hematologic/immune diseases); and (5) loss to follow-up.

**Figure 1:**
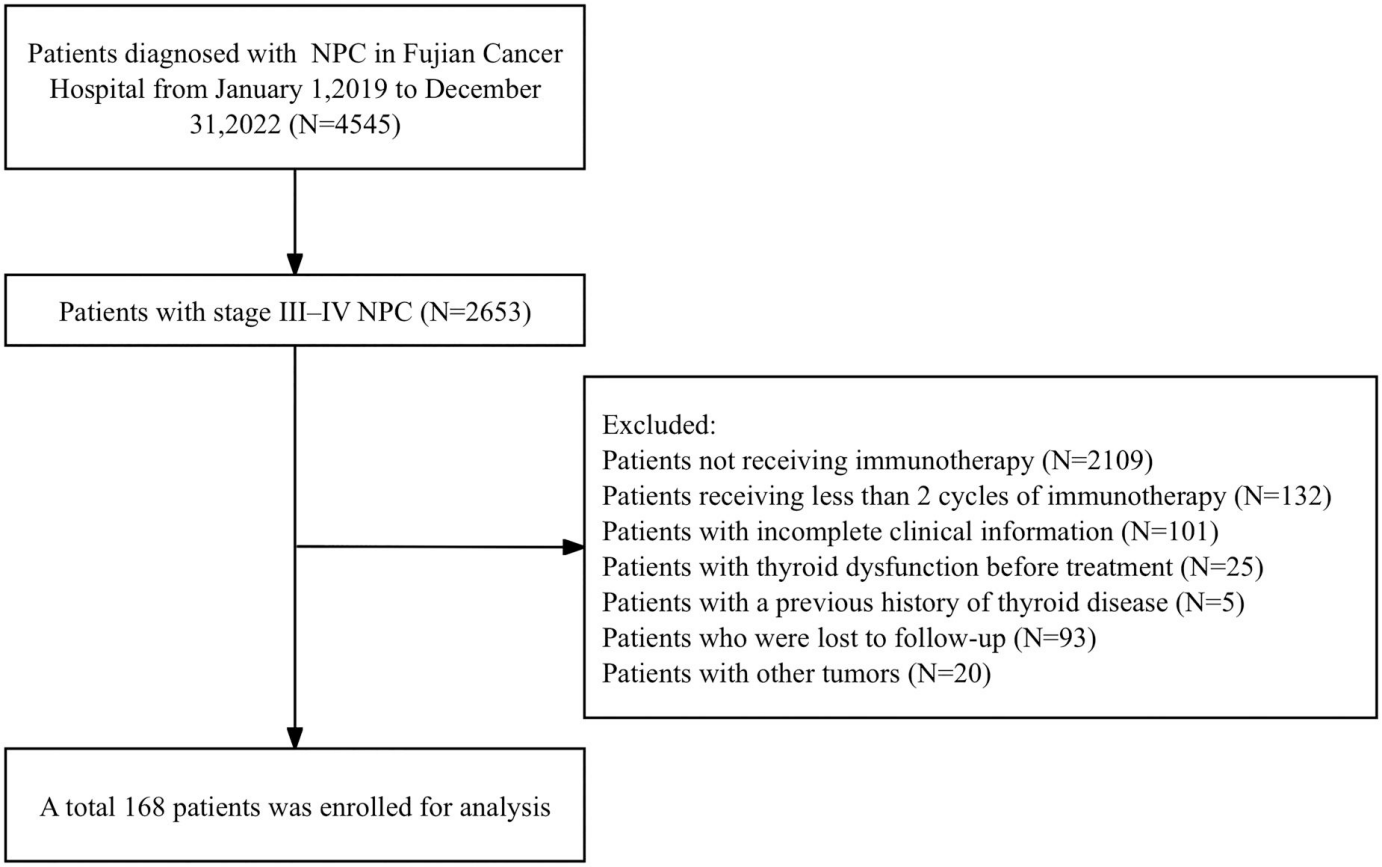
The flowchart of the patient selection.

This study was approved by the institutional ethics committees and conducted in accordance with the Declaration of Helsinki. Due to its retrospective nature, informed consent was waived. After screening, 168 eligible patients receiving PD-1 therapy were included in the final analysis (Figure 1).

### Treatment and follow-up

Patients received PD-1 inhibitors every three weeks, either as monotherapy or combined with chemotherapy or targeted therapy. Agents included camrelizumab, tislelizumab, sintilimab, pembrolizumab, toripalimab, and nivolumab. Standard doses were: 200 mg for camrelizumab, tislelizumab, sintilimab, penpulimab, and pembrolizumab; 240 mg for toripalimab; and 360 mg for nivolumab. Treatment continued until disease progression, unacceptable toxicity, clinical deterioration, or patient withdrawal.

Patients underwent routine clinical and laboratory evaluations before each treatment cycle. Baseline data included demographics, BMI, TNM staging (restaged according to the 8th edition of the American Joint Committee on Cancer), ECOG score, Epstein–Barr virus DNA (EBV-DNA) levels, immunotherapy regimen and number of cycles, beginning phase, hematologic indices, biochemical parameters, and coagulation profiles. Plasma EBV-DNA was measured using a real-time quantitative polymerase chain reaction (qPCR) assay targeting the BamHI-W region of the EBV genome.^23^^,^^24^

After treatment, patients were followed every 3 months for 2 years, twice annually for the next 3 years, and annually thereafter. Survival and tumor status were assessed via clinical records and telephone follow-up. Efficacy was evaluated per RECIST v1.1. Suspected progression was pathologically confirmed when feasible. For inaccessible lesions with characteristic imaging features, diagnosis relied on at least two imaging modalities, irrespective of symptoms. PFS was defined from the first PD-1 dose to progression, death, or study cutoff. OS was defined from treatment initiation to death or cutoff.

### Assessment of study variables

Thyroid function was routinely monitored by measuring serum thyroid-stimulating hormone (TSH), free thyroxine (FT4), and free triiodothyronine (FT3) at baseline (within one month prior to treatment initiation) and before each subsequent treatment cycle. The diagnosis of trTD was primarily based on abnormalities in laboratory test results, and the specific diagnostic criteria were defined according to previously published studies:^20^^,^^25–27^

- Non-trTD: TSH and FT4 remained within institutional reference ranges (TSH: 0.34-5.60 mIU/L; FT4: 7.98-19.24 pmol/L) throughout treatment.
- TrTD: Development of hyperthyroidism, hypothyroidism (clinical or subclinical), or biphasic thyroid dysfunction. Hyperthyroidism was defined as a TSH level below the lower reference limit with normal and elevated FT4; clinical hypothyroidism was characterized by an elevated TSH level along with a decreased FT4 level; subclinical hypothyroidism was defined as a TSH level greater than 10 mIU/L with normal FT4 and biphasic thyroid dysfunction was defined as the occurrence of hyperthyroidism followed by hypothyroidism or vice versa during treatment.

FT3 (reference: 3.53-7.37 pmol/L) was also monitored to support diagnosis and dynamic assessment. TrTD severity was graded per CTCAE v5.0 (Grades 1-5). Assessments were made by treating physicians or clinical pharmacists. Other irAEs were defined as new-onset events post-immunotherapy, confirmed by pathology or clinical diagnosis, after excluding other causes such as chemotherapy-related toxicity.

### Statistical analysis

Statistical analyses were conducted using R (v4.4.1) and SPSS (v26.0). Continuous variables were compared with Student’s *t*-test or Mann–Whitney *U* test. Categorical variables with chi-square or Fisher’s exact test. Receiver operating curve (ROC) was used to identify optimal cutoffs (Youden index). Logistic regression was used to identify risk factors for trTD. Survival analyses were performed using Kaplan–Meier curves and Cox proportional hazards models. Spearman correlation evaluated associations between immunotherapy cycles and thyroid indices. A predictive nomogram for trTD was developed using significant variables, and its performance assessed by the area under the ROC curve (AUC) and calibration plots. A two-sided *P* < .05 indicated statistical significance.

## Results

### Patient characteristics

A total of 168 patients were enrolled in this cohort, including 132 males and 36 females, with a median age of 47 years (interquartile range [IQR], 39-55 years). Among them, 32 patients (19.05%) were classified as stage III, 67 (39.88%) as stage IVA, and 69 (41.07%) as stage IVB. Treatment regimens included chemotherapy combined with PD-1 inhibitors (*n* = 2), radiotherapy plus PD-1 inhibitors (*n* = 115), and concurrent chemoradiotherapy with PD-1 inhibitors (*n* = 51). The median follow-up duration was 32 months (IQR, 25.75-40 months). The median PFS was 27.00 months (IQR, 18.75-35 months), and the median OS was 28.50 months (IQR, 24-38 months).

Among the entire cohort, 83 individuals (49.40%) developed trTD, while the remaining 85 patients (50.60%) maintained non-trTD group. In the trTD group, clinical hypothyroidism occurred in 17 patients (20.48%), subclinical hypothyroidism in 7 (8.43%), hyperthyroidism in 34 (40.96%), and biphasic thyroid dysfuction in 25 (30.12%). The majority of thyroid dysfunctions were mild (grades 1-2: 82/83, 98.80%), with only one case (1.20%) of grade 3 toxicity and no grades 4-5 events observed.

As shown in Table 1, there were no significant differences between the trTD and non-trTD groups in terms of age, T/N/M stage, clinical stage, treatment, beginning phase, BMI, ECOG performance status, EBV DNA levels, PT, or LDH. However, the trTD group had a significantly higher proportion of female patients (*P* = .012), and those with NLR > 7.71 (*P* = .045), ALB > 39.45 g/L (*P* = .020), ALT > 16.50 U/L (*P* = .023), and TBIL > 8.45 μmol/L (*P* = .013).

**Table 1:** Baseline characteristics of patients with advanced NPC before PD-1 inhibitor therapy.

Variable	Non-trTD	TrTD	*P*
***n***	85	83	
**Age(years) (*n*, %)**			1
** >60**	9 (10.6)	9 (10.8)	
** ≤60**	76 (89.4)	74 (89.2)	
**Gender (*n*, %)**			.012
** Female**	11 (12.9)	25 (30.1)	
** Male**	74 (87.1)	58 (69.9)	
***T* (*n*, %)**			.263
** 1**	9 (10.5)	14 (16.9)	
** 2**	10 (11.8)	7 (8.4)	
** 3**	35 (41.2)	41 (49.4)	
** 4**	31 (36.5)	21 (25.3)	
***N* (*n*, %)**			.43
** 0**	3 (3.5)	1 (1.2)	
** 1**	24 (28.2)	18 (21.7)	
** 2**	24 (28.2)	22 (26.5)	
** 3**	34 (40.1)	42 (50.6)	
***M* (*n*, %)**			.618
** 0**	48 (56.5)	51 (61.4)	
** 1**	37 (43.5)	32 (38.6)	
**Eighth AJCC stage (*n*, %)**			.263
** III**	19 (22.4)	13 (15.7)	
** IVA**	29 (34.1)	38 (45.8)	
** IVB**	37 (43.5)	32 (38.5)	
**Treatment (*n*, %)**			.328
** C + I**	2 (2.4)	0 (0.0)	
** RT + I**	56 (65.8)	59 (71.1)	
** CCRT + I**	27 (31.8)	24 (28.9)	
**Drugs (*n*, %)**			.753
** Camrelizumab**	10 (11.8)	7 (8.4)	
** Nivolumab**	2 (2.4)	3 (3.6)	
** Pembrolizumab**	6 (7.1)	8 (9.6)	
** Penpulimab**	1 (1.2)	0 (0.0)	
** Sintilimab**	5 (5.9)	2 (2.4)	
** Tislelizumab**	12 (14.0)	14 (16.9)	
** Toripalimab**	49 (57.6)	49 (59.1)	
**Beginning phase (*n*, %)**			.596
** After RT**	23 (27.1)	17 (20.5)	
** Before RT**	48 (56.5)	52 (62.7)	
** During RT**	14 (16.5)	14 (16.9)	
**BMI (kg/m^2^) (*n*, %)**			.787
** >21.35**	60 (70.6)	56 (67.5)	
** ≤21.35**	25 (29.4)	27 (32.5)	
**ECOG performance status (*n*, %)**			.764
** >1**	19 (22.4)	16 (19.3)	
** ≤1**	66 (77.6)	67 (80.7)	
**EBVDNA (copies/mL) ( *n*, %)**			.63
** >1925**	45 (52.9)	48 (57.8)	
** ≤1925**	40 (47.1)	35 (42.2)	
**PT (s) (*n*, %)**			.942
** >12.45**	25 (29.4)	23 (27.7)	
** ≤12.45**	60 (70.6)	60 (72.3)	
**INR (*n*, %)**			.532
** >0.975**	43 (50.6)	37 (44.6)	
** ≤0.975**	42 (49.4)	46 (55.4)	
**APTT (s) (*n*, %)**			.545
** >34.2**	36 (42.4)	40 (48.2)	
** ≤34.2**	49 (57.6)	43 (51.8)	
**FIB(g/L) (*n*, %)**			.089
** >3.24**	57 (67.1)	44 (53.0)	
** ≤3.24**	28 (32.9)	39 (47.0)	
**TT (s) (*n*, %)**			.182
** >7.15**	78 (91.8)	81 (97.6)	
** ≤7.15**	7 (8.2)	2 (2.4)	
**Ddimmer (µg/mL); *n*, %)**			.518
** >0.46**	39 (45.9)	33 (39.8)	
** ≤0.46**	46 (54.1)	50 (60.2)	
**PLT (10^9^/L) (*n*, %)**			.403
** >260**	35 (41.2)	28 (33.7)	
** ≤260**	50 (58.8)	55 (66.3)	
**WBC (10^9^/L) (*n*, %)**			.139
** >10.00**	16 (18.8)	8 (9.6)	
** ≤10.00**	69 (81.2)	75 (90.4)	
**NEUT (10^9^/L) (*n*, %)**			.46
** >8.90**	10 (11.8)	6 (7.2)	
** ≤8.90**	75 (88.2)	77 (92.8)	
**LYMPH (10^9^/L) (*n*, %)**			.507
** >0.87**	64 (75.3)	67 (80.7)	
** ≤0.87**	21 (24.7)	16 (19.3)	
**MONO (10^9^/L) (*n*, %)**			.919
** >0.32**	60 (70.6)	57 (68.7)	
** ≤0.32**	25 (29.4)	26 (31.3)	
**EO (10^9^/L) (*n*, %)**			1
** >0.18**	18 (21.2)	18 (21.7)	
** ≤0.18**	67 (78.8)	65 (78.3)	
**PLR (*n*, %)**			.157
** >148.25**	61 (71.8)	50 (60.2)	
** ≤148.25**	24 (28.2)	33 (39.8)	
**NLR (*n*, %)**			.045
** >7.71**	5 (5.9)	14 (16.9)	
** ≤7.71**	80 (94.1)	69 (83.1)	
**MLR (*n*, %)**			.182
** >0.42**	35 (41.2)	25 (30.1)	
** ≤0.42**	50 (58.8)	58 (69.9)	
**ALB (g/L) (*n*, %)**			.02
** >39.45**	48 (56.5)	62 (74.7)	
** ≤39.45**	37 (43.5)	21 (25.3)	
**ALT (U/L) (*n*, %)**			.023
** >16.5**	53 (62.4)	66 (79.5)	
** ≤16.5**	32 (37.6)	17 (20.5)	
**AST(U/L) (*n*, %)**			.939
** >34**	16 (18.8)	17 (20.5)	
** ≤34**	69 (81.2)	66 (79.5)	
**LDH(U/L) (*n*, %)**			.484
** >248**	19 (22.4)	14 (16.9)	
** ≤248**	66 (77.6)	69 (83.1)	
**Glu(mmol/L) (*n*, %)**			.784
** >4.86**	57 (67.1)	53 (63.9)	
** ≤4.86**	28 (32.9)	30 (36.1)	
**ALP(U/L) (*n*, %)**			.456
** >113**	17 (20.0)	12 (14.5)	
** ≤113**	68 (80.0)	71 (85.5)	
**TBIL(μmol/L) (*n*, %)**			.013
** >8.45**	58 (68.2)	71 (85.5)	
** ≤8.45**	27 (31.8)	12 (14.5)	
**TC (mmol/L) (*n*, %)**			.099
** >5.80**	28 (32.9)	17 (20.5)	
** ≤5.80**	57 (67.1)	66 (79.5)	
**TG (mmol/L) (*n*, %)**			.145
** >0.89**	78 (91.8)	69 (83.1)	
** ≤0.89**	7 (8.2)	14 (16.9)	
**HDL (mmol/L) (*n*, %)**			1
** >2.00**	2 (2.4)	2 (2.4)	
** ≤2.00**	83 (97.6)	81 (97.6)	
**LDL (mmol/L) (*n*, %)**			.243
** >3.58**	35 (41.2)	26 (31.3)	
** ≤3.58**	50 (58.8)	57 (68.7)	
**FT3 (pmol/L) (*n*, %)**			.111
** >5.52**	22 (25.9)	32 (38.6)	
** ≤5.52**	63 (74.1)	51 (61.4)	
**FT4 (pmol/L) (*n*, %)**			.065
** >12.06**	35 (41.2)	47 (56.6)	
** ≤12.06**	50 (58.8)	36 (43.4)	
**TSH (mIU/L) (*n*, %)**			.076
** >4.186**	3 (3.5)	10 (12.0)	
** ≤4.186**	82 (96.5)	73 (88.0)	

Abbreviations: C + I, Chemotherapy + Immunotherapy; RT + I, Radiotherapy + Immunotherapy.

CCRT + I, Concurrent Chemoradiotherapy + Immunotherapy.

### Prognostic value of trTD in advanced NPC

**Figure 2:**
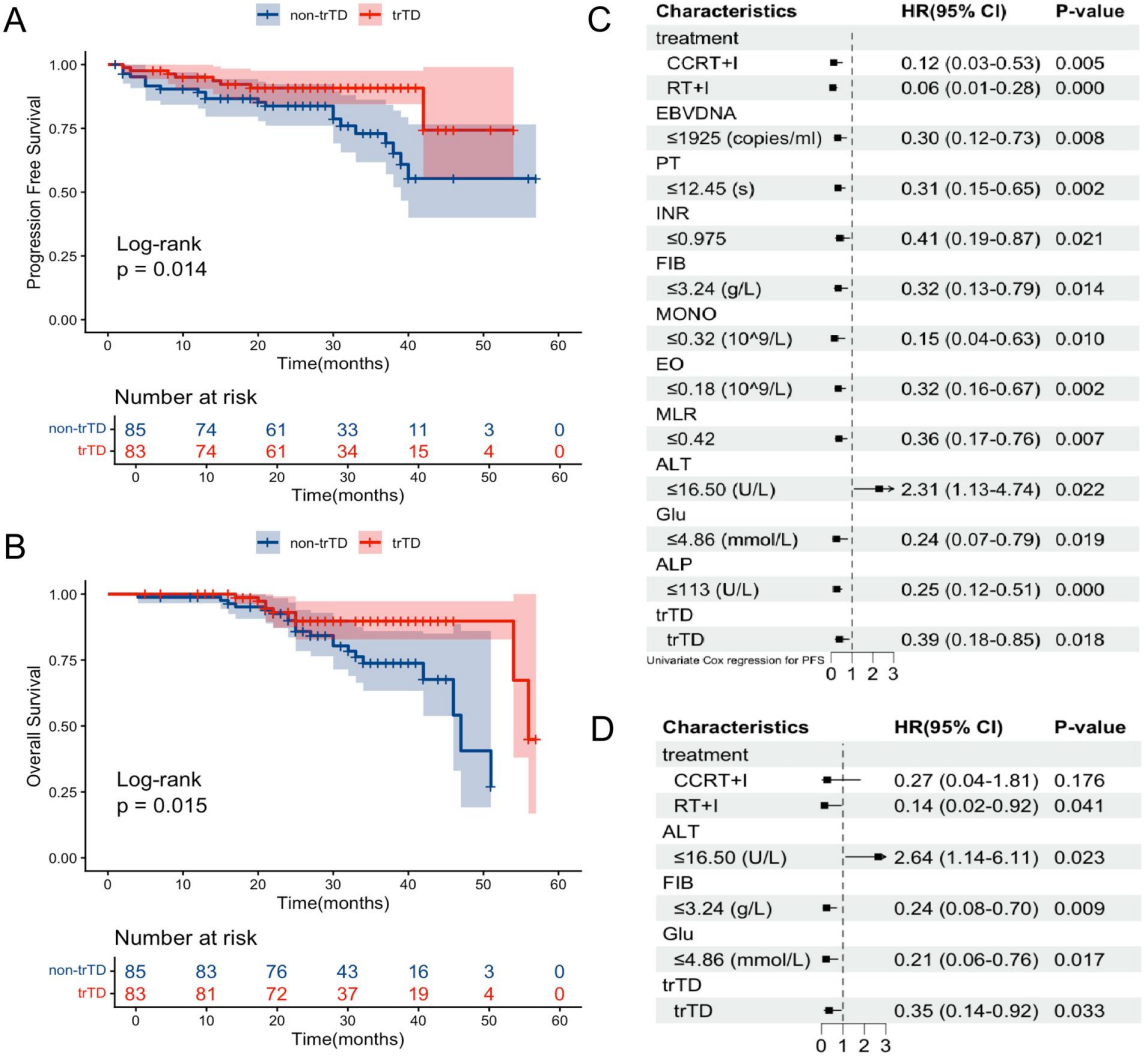
Prognostic significance of trTD in PD-1 inhibitor-treated advanced NPC. (A) PFS by trTD status. (B) OS by trTD status. Kaplan–Meier curves with log-rank test *P*-values are shown for all survival analyses. (C) Univariate and (D) multivariate Cox regression analyses of factors associated with PFS. Hazard ratios with 95% confidence intervals are shown. Abbreviations: CI, confidence interval; HR, hazard ratio; NPC, nasopharyngeal carcinoma; OS, overall survival; PFS, progression-free survival; trTD, treatment-related thyroid dysfunction.

In this cohort, K-M survival analysis showed that patients who developed trTD had significantly longer PFS and OS compared to those without trTD, indicating that trTD was associated with favorable clinical outcomes (Figure 2A and B). Further subgroup analysis revealed that the different trTD phenotypes—including clinical hypothyroidism, subclinical hypothyroidism, hyperthyroidism, and biphasic thyroiditis—were not significantly associated with differences in survival outcomes (Figures S1 and S2). Univariate and multivariate Cox regression analyses were subsequently conducted to explore independent prognostic factors. For PFS, treatment, ALT, fibrinogen (FIB), glucose (Glu), and trTD were identified as independent prognostic indicators (Figure 2C and D). For OS, ALT, EBV-DNA levels, Glu, and treatment emerged as independent prognostic factors (Figure S3). Notably, trTD remained an independent predictor for improved PFS (*P* = .033), suggesting its potential utility as a prognostic biomarker for patients with advanced NPC receiving PD-1 inhibitor therapy.

### Identification of risk factors for trTD and nomogram construction

**Figure 3:**
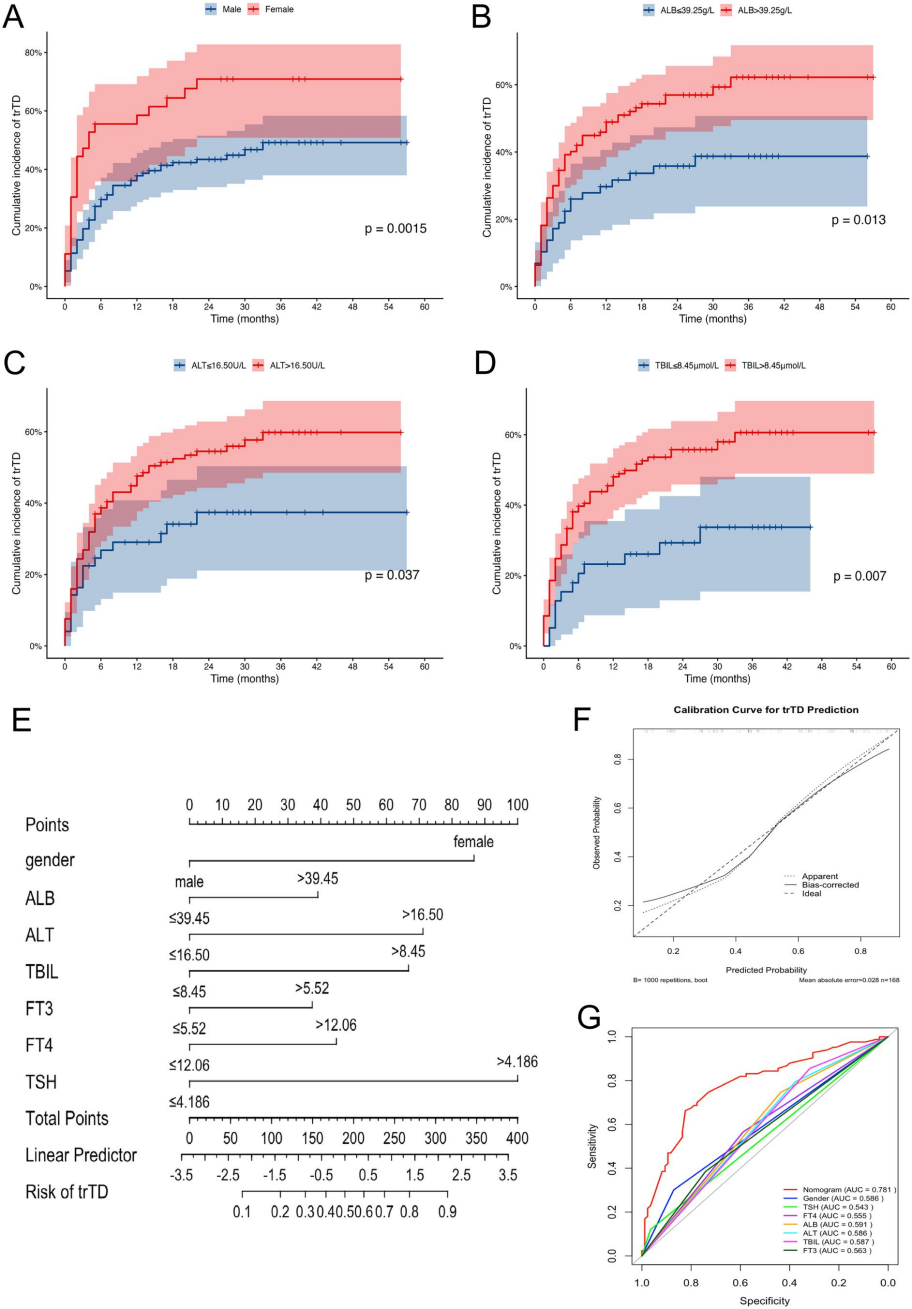
Risk factor analysis and nomogram development for predicting trTD in PD-1 inhibitor-treated patients. (A-D) Cumulative incidence of trTD stratified by: (A) Gender, (B) ALB, (C) ALT, and (D) TBIL. (E) Nomogram model established by independent risk factors. (F) The calibration curves for (Continued)validation of the nomogram. (G)The ROC curves comparing the accuracy of model and the other risk factors for predicting. Abbreviations: ALB, albumin; ALT, alanine aminotransferase; AUC, area under the curve; ROC, receiver operating characteristic; TBIL, total bilirubin, NLR, neutrophil-to-lymphocyte ratio; trTD, treatment-related thyroid dysfunction.

To identify risk factors associated with the development of trTD, we conducted both univariate and multivariate logistic regression analyses. As shown in Table 2, univariate analysis revealed that gender, NLR, ALB, ALT, TBIL, and FT4 levels were significantly associated with trTD. In the multivariate model, female (OR = 4.15, 95% CI: 1.66-10.38, *P* = .002), pretreatment ALB > 39.45 g/L (OR = 2.60, 95% CI: 1.18-5.72, *P* = .017), ALT > 16.50 U/L (OR =3.10, 95% CI: 1.38-6.97, *P* = .006), and TBIL > 8.45 µmol/L (OR = 2.92, 95% CI: 1.18-7.25, *P* = .021) remained independent predictors of the occurrence of trTD. These findings indicate that the above parameters serve as independent risk factors for trTD during PD-1 therapy. Cumulative incidence plots further confirmed that patients with these risk factors—female, elevated ALB, ALT, and TBIL—had a significantly higher likelihood of developing trTD (Figure 3A-D). Given the prognostic value of trTD and the identification of these risk factors, we developed a nomogram to predict the probability of trTD occurrence in patients receiving PD-1 therapy. We incorporated the previously identified variables—gender, baseline ALB, ALT, TBIL, and key baseline thyroid function indicators (FT3, FT4, TSH)—into the nomogram (Figure 3E). The model enabled individualized risk prediction, and the calibration plot showed excellent agreement between predicted and observed outcomes (Figure 3F). Furthermore, ROC analysis demonstrated that the nomogram outperformed any individual factor, achieving an AUC of 0.781 (Figure 3G), highlighting its robust predictive performance.

**Table 2:** Univariate and multivariate analysis of treatment-related thyroid dysfunction (trTD) with Logistic regression models.

Variable	Univariate analysis			Multivariate analysis		
	OR	95% CI	*P*	OR	95% CI	*P*
**Gender**			.008			.002
** Male**	Reference			Reference		
** Female**	2.90	1.32-6.38		4.15	1.66-10.38	
**NLR**			.031			.055
** ≤7.71**	Reference			Reference		
** >7.71**	3.25	1.11-9.47		3.18	0.98-10.33	
**ALB**			.014			.017
** ≤39.45 g/L**	Reference			Reference		
** >39.45 g/L**	2.28	1.18-4.38		2.60	1.18-5.72	
**ALT**			.016			.006
** ≤16.50 U/L**	Reference			Reference		
** >16.50 U/L**	2.34	1.18-4.68		3.10	1.38-6.97	
**TBIL**			.009			.021
** ≤8.45 μmol/L**	Reference			Reference		
** >8.45 μmol/L**	2.75	1.28-5.91		2.92	1.18-7.25	
**FT4**			.046			.081
** ≤12.06 pmol/L**	Reference			Reference		
** >12.06 pmol/L**	1.87	1.01-3.44		1.88	0.93-3.80	

### Dynamic association between trTD and PD-1 therapy duration

**Figure 4:**
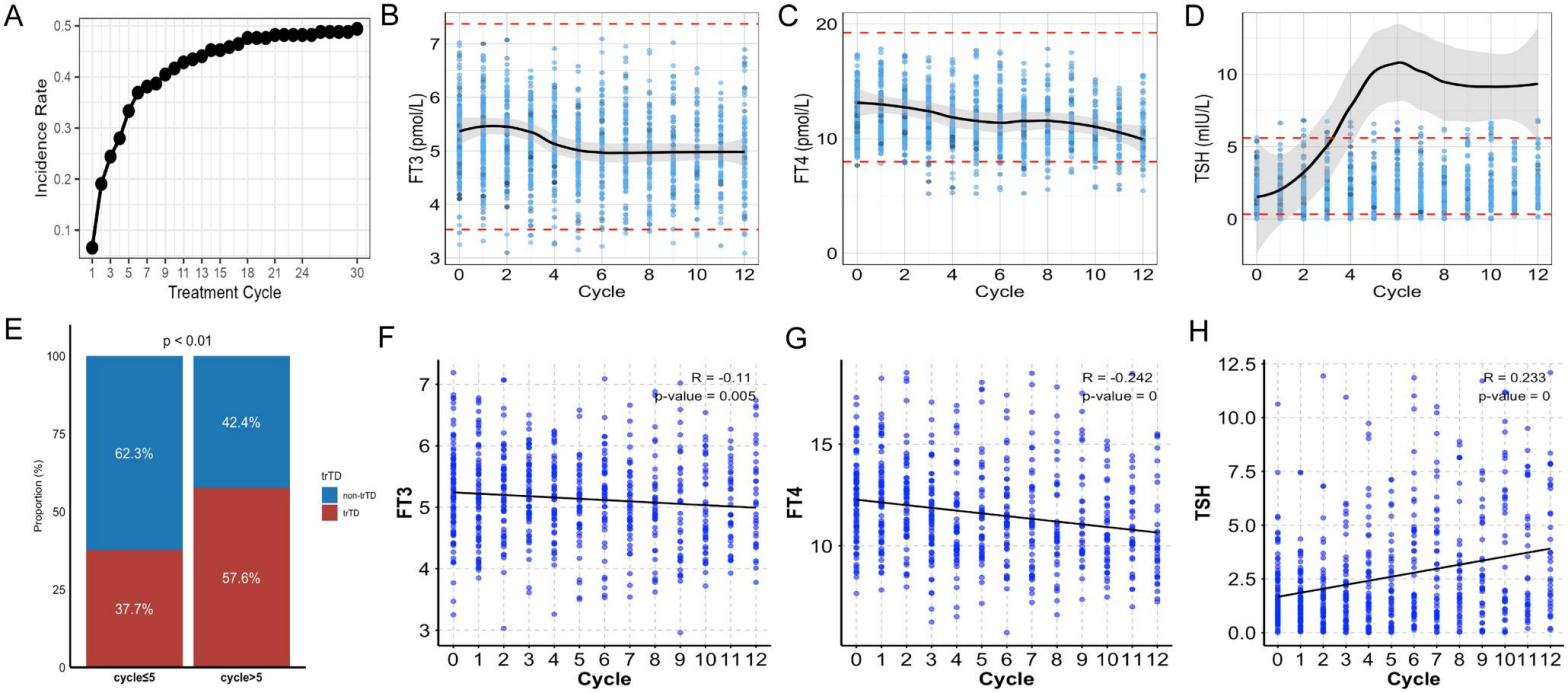
Dynamic Association Between trTD Incidence, Thyroid Function, and PD-1 Therapy. (A) The trend of trTD incidence with the number of cycles. (B-D) The change trend of free triiodothyronine (FT3; normal range: 3.53-7.37 pmol/L), free thyroxine (FT4; normal range: 7.98-19.24 pmol/L), and thyroid-stimulating hormone (TSH; normal range: 0.34-5.60 mIU/L) with the increase of cycle number. (E) Proportion differences of trTD and non- trTD between short (≤5) and long (>5) treatment cycles. (F-H) Spearman correlation analysis between thyroid function (FT3, FT4, TSH) and immunotherapy cycles.

To further explore the relationship between trTD and immunotherapy exposure time, we conducted a dynamic analysis of trTD incidence across treatment cycles. The incidence of trTD increased progressively with treatment cycles and plateaued around the 18th cycle, with a cumulative incidence approaching 50% (Figure 4A). Additionally, we examined changes in thyroid function indicators during immunotherapy. FT3 and FT4 levels showed a gradual decline, while TSH levels rose sharply and stabilized after the fourth cycle (Figure 4B-D). Notably, FT3 and FT4 remained within the reference range, while TSH exceeded the upper limit (>10 mIU/L) after cycle 4, indicating that TSH may be a more sensitive early marker for trTD development. Patients receiving more than five cycles of immunotherapy were more likely to develop trTD (Figure 4E). In the trTD subgroup, FT3 and FT4 levels were negatively correlated with treatment duration, while TSH was positively correlated (Figure 4F-H). All associations were statistically significant (*P* < .05). This observation warrants further investigation into the underlying mechanisms of trTD.

## Discussion

NPC is an aggressive cancer with a distinct geographic prevalence.^1^ In recent years, immune checkpoint inhibitors, particularly PD-1 inhibitors, have emerged as an important therapeutic option for patients with advanced NPC.^4^^,^^28^ However, the clinical significance of irAEs, particularly thyroid dysfuction, in terms of treatment tolerability, long-term management, and prognostic assessment, remains to be fully elucidated.^5^^,^^29^ This study systematically evaluated the incidence, clinical characteristics, predictive factors, and prognostic implications of trTD in patients with advanced NPC receiving PD-1 inhibitor therapy. Our results revealed that approximately half of the patients developed trTD during immunotherapy, with female and elevated baseline levels of ALB, ALT, and TBIL identified as independent predictors. A nomogram incorporating these variables together with thyroid function indices exhibited good discriminative performance. Importantly, the occurrence of trTD was strongly associated with improved PFS and OS, suggesting that trTD may serve as a potential prognostic biomarker for immunotherapy benefit in advanced NPC.

In our cohort of 168 patients with advanced NPC, 49.4% developed trTD, manifesting as clinical or subclinical hypothyroidism, hyperthyroidism, or biphasic thyroid dysfunction. Most events were low-grade (CTCAE grades 1-2), consistent with previous reports of generally mild trTD. Notably, this incidence is markedly higher than that reported in other solid tumor populations. Prior evidence indicates that approximately 10%-30% of patients with non-small cell lung cancer, melanoma, and renal cell carcinoma receiving immune checkpoint inhibitor therapy develop thyroid dysfunction.^30–32^ In clinical trials of patients with recurrent or metastatic head and neck squamous cell carcinoma, the incidence of thyroid dysfunction was typically 8%-16%.^33^^,^^34^ In contrast, the markedly elevated incidence observed in advanced NPC suggests a unique susceptibility in this population. This difference is likely multifactorial. First, most patients with advanced NPC have previously undergone head and neck radiotherapy, which has been confirmed as a significant risk factor for thyroid dysfunction.^35^ Radiation can directly damage thyroid follicular epithelial cells, disrupt microvascular structures, and induce local chronic inflammation, thereby impairing thyroid hormone synthesis and secretion.^36^^,^^37^ Radiotherapy may also alter the exposure of thyroid antigens, potentially triggering or exacerbating immune-mediated thyroid injury.^38^ As an endocrine organ rich in self-antigens, the thyroid is particularly sensitive to immune dysregulation, making it a common target of irAEs.^39^ Moreover, radiotherapy may synergize with immunotherapy by promoting antigen release, enhancing antigen presentation, and reshaping the local immune microenvironment, thereby amplifying immunotherapeutic effects while potentially increasing autoimmune toxicity.^40^^,^^41^ Consistently, the CONTINUUM study reported a thyroid dysfunction incidence of 46.8% in patients with locally advanced NPC receiving immunotherapy, closely aligning with our findings.^8^

As clinical evidence supporting PD-1 blockade in NPC continues to accumulate, the underlying immunological mechanisms are becoming increasingly clear.^4^^,^^28^ By inhibiting the PD-1/PD-L1 axis, PD-1 inhibitors relieve T-cell suppression in the tumor microenvironment, restoring the function of exhausted CD8^+^T cells in EBV-associated NPC, including enhanced proliferation and cytotoxicity.^42^^,^^43^ Given the strong association of NPC with EBV infection and the high PD-L1 expression in tumor cells, PD-1 blockade effectively augments EBV antigen-driven antitumor immunity, thereby improving patient outcomes. Increasing evidence indicates that the occurrence of immunotherapy-related thyroid dysfunction is positively correlated with treatment efficacy.^44–46^ For example, a real-world study of Chinese lung cancer patients reported significantly longer overall survival among those who developed thyroid dysfunction, suggesting its potential as a prognostic biomarker.^47^ In this study, multivariate Cox regression analysis also identified trTD as an independent prognostic factor for PFS, consistent with observations in gastric cancer and renal cell carcinoma.^48^^,^^49^ In predictive factor analysis, female sex and elevated baseline ALB, ALT, and TBIL were independently associated with trTD development. Prior studies reported higher trTD incidence in females, potentially due to sex-related immune differences and higher baseline prevalence of autoimmune thyroid disease in women.^50^^,^^51^ The association between elevated ALB and trTD risk has also been documented.^15^^,^^52–54^ Albumin plays a critical role in thyroid hormone transport, and its levels may influence free hormone bioavailability via alterations in thyroxine-binding affinity.^55^^,^^56^ Notably, we observed for the first time a significant association between the number of immunotherapy cycles and trTD risk. Patients receiving more than 5 cycles of PD-1 inhibitors exhibited markedly increased trTD risk. Time-dependent analysis revealed a pronounced rise in trTD incidence around the fifth cycle, characterized by TSH elevation and progressive declines in FT3 and FT4. The risk demonstrated a time- and dose-dependent pattern, plateauing after approximately 18 cycles, suggesting delayed thyroid dysfunction may result from sustained immune activation, progressive regulatory T-cell impairment, and gradual loss of peripheral tolerance. Conversely, patients without trTD in the first 18 cycles may reflect insufficient immune activation or inherent resistance, correlating with poorer prognosis.

Dynamics inform thyroid monitoring strategies during immunotherapy. According to ESMO guidelines, thyroid function should be assessed every cycle during the first three months, followed by alternate-cycle monitoring.^27^ Our findings suggest that for low-risk patients who remain trTD-free beyond 18 cycles, extended monitoring intervals may be reasonable, pending prospective validation. Furthermore, dynamic TSH monitoring appears to provide a sensitive and clinically practical approach for early trTD detection. The nomogram developed here allows quantitative risk stratification, guiding individualized surveillance. High-risk patients should continue to undergo close monitoring, particularly after the fifth treatment cycle, whereas low-risk and stable patients may have extended monitoring intervals. More frequent surveillance and timely endocrine intervention in high-risk patients may help prevent treatment interruption and reduce cardiovascular risk. Interestingly, we observed that patients receiving more than five cycles of immunotherapy had a significantly higher risk of trTD, a novel finding not previously reported. This may be related to prolonged immune activation and cumulative toxicity with extended PD-1 blockade. Temporal analysis revealed a surge in trTD incidence around the fifth cycle, marked by rising TSH levels (approaching 10 mIU/L) and gradual declines in FT3 and FT4 levels. At the same time, a dose-dependent increase in trTD incidence was observed, leveling off after approximately 18 cycles. This pattern suggests that patients who do not develop trTD within the first 18 cycles may have a poorer prognosis, possibly indicating immune resistance. These dynamics suggest delayed-onset thyroiditis likely driven by progressive Treg dysfunction and loss of peripheral tolerance. While previous studies emphasized the role of baseline thyroid antibodies,^22^ our findings suggest that dynamic monitoring—particularly of TSH—may offer a more practical and sensitive approach to early trTD detection.

Nevertheless, this study has limitations. As a single-center retrospective analysis, generalizability may be limited and selection bias possible. The small number of patients with certain trTD subtypes constrained subtype-specific survival analyses. External validation of the predictive nomogram is lacking, and thyroid autoantibody status and radiation dose parameters were not systematically collected. Future multicenter, prospective studies integrating multi-omics approaches, such as single-cell or spatial transcriptomics, are needed to further elucidate trTD mechanisms and optimize management in high-risk patients.

In conclusion, this study highlights the high incidence of trTD in advanced NPC patients receiving PD-1 inhibitors and its strong association with survival benefit. a practical nomogram was constructed to enable early risk assessment and individualized monitoring. These findings provide critical guidance for trTD surveillance and precision management in the era of NPC immunotherapy.

## Conclusion

This study systematically reveals the high incidence of trTD and its significant association with improved survival in patients with advanced NPC treated with PD-1 inhibitors. The risk of trTD increased with the accumulation of treatment cycles, and TSH level increased significantly after the fifth cycle of treatment, suggesting that it can be used as an early warning indicator. Female, and baseline parameters (high ALB, ALT, and TBIL) were independent predictors of trTD. The nomogram model based on these factors and thyroid indicators provided a practical tool for clinical identification of high-risk patients. This study provides a foundation for risk stratification of trTD in NPC, paving the way for personalized immunotherapy monitoring strategies.

## Supplementary Material

oyag066_Supplementary_Data

## Data Availability

The data underlying this article cannot be shared publicly due to the privacy of individuals that participated in the study. The data will be shared on reasonable request to the corresponding author.
