# Supplementary material for: Predictive modeling of treatment-related thyroid dysfunction and prognostic implication in advanced nasopharyngeal carcinoma with PD-1 inhibitors
Source: Oncologist. 2026 Feb 25;31(4):oyag066. doi: 10.1093/oncolo/oyag066 (PMC13026421; doi:10.1093/oncolo/oyag066)

**Supplementary Figure 1. Kaplan-Meier survival analysis of progression-free survival in the trTD subgroups.**(A)Biphasic thyroid dysfunction vs Clinical hypothyroidism; (B) Subclinical hypothyroidism vs Hyperthyroidism; (C) Biphasic thyroid dysfunction vs Subclinical hypothyroidism; (D) Clinical hypothyroidism vs Hyperthyroidism; (E) Clinical hypothyroidism vs Subclinical hypothyroidism; (F)Biphasic thyroid dysfunction vs Hyperthyroidism.


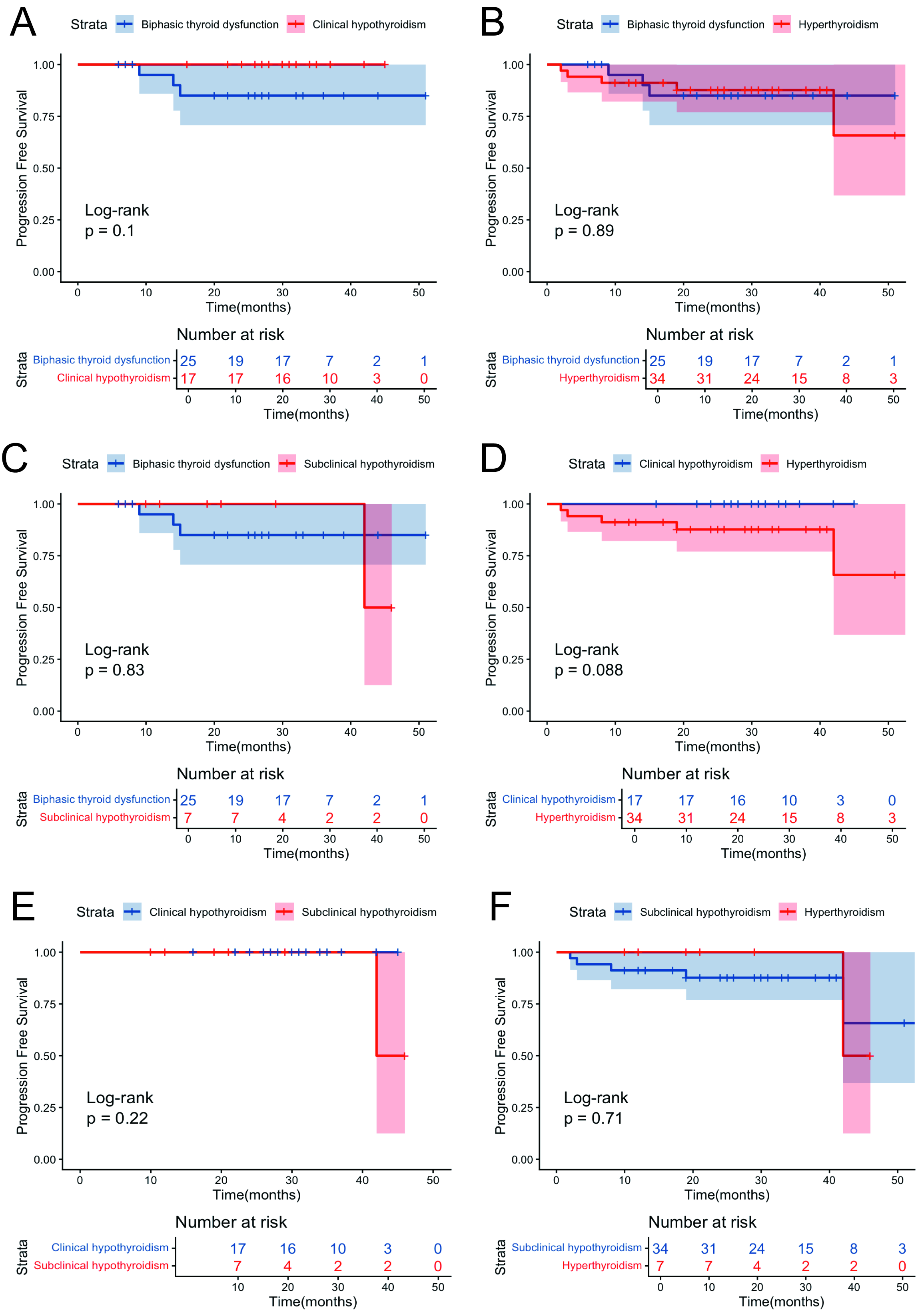


**Supplementary Figure 2. Kaplan-Meier survival analysis of overall survival in the trTD subgroups.** (A)Biphasic thyroid dysfunction vs Clinical hypothyroidism; (B) Subclinical hypothyroidism vs Hyperthyroidism; (C) Biphasic thyroid dysfunction vs Subclinical hypothyroidism; (D) Clinical hypothyroidism vs Hyperthyroidism; (E) Clinical hypothyroidism vs Subclinical hypothyroidism; (F)Biphasic thyroid dysfunction vs Hyperthyroidism.


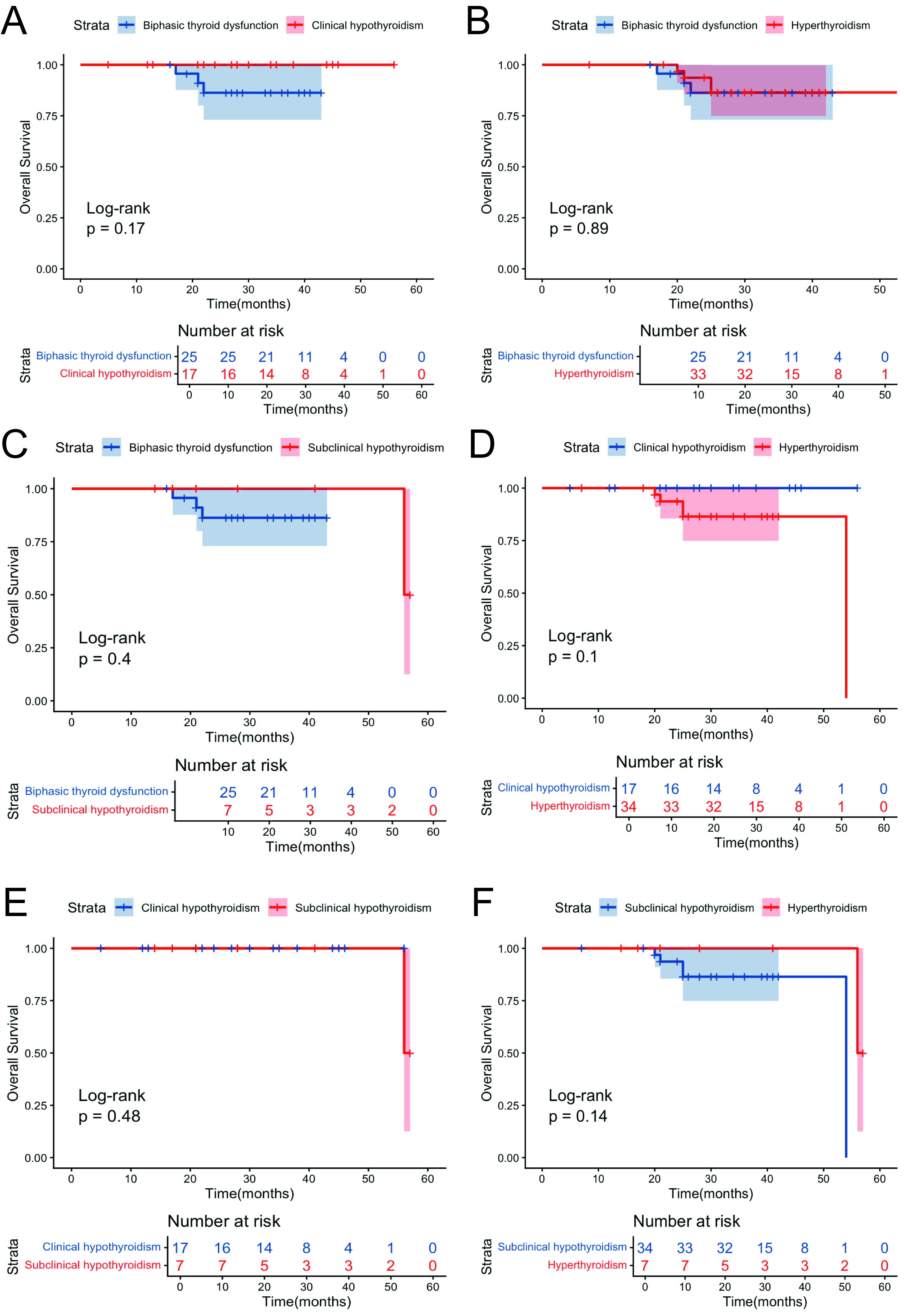


**Supplementary Figure 3.** Univariate (A) and multivariate (B) cox regression analyses of overall survival prognostic factors in advanced nasopharyngeal carcinoma patients receiving PD-1 inhibitors.


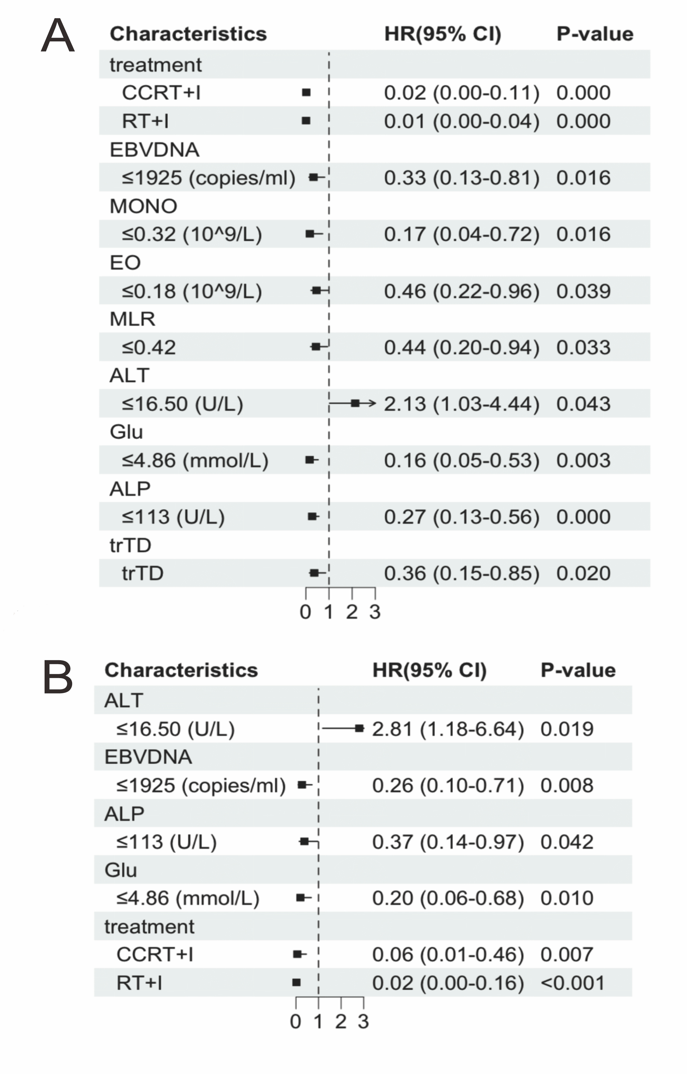

Supplement: oyag066_Supplementary_Data [file oyag066_supplementary_data.zip › Supplementary Figures.docx]
