# Supplementary figures and images for: Predictive modeling of treatment-related thyroid dysfunction and prognostic implication in advanced nasopharyngeal carcinoma with PD-1 inhibitors
Source: Oncologist. 2026 Feb 25;31(4):oyag066. doi: 10.1093/oncolo/oyag066 (PMC13026421; doi:10.1093/oncolo/oyag066)

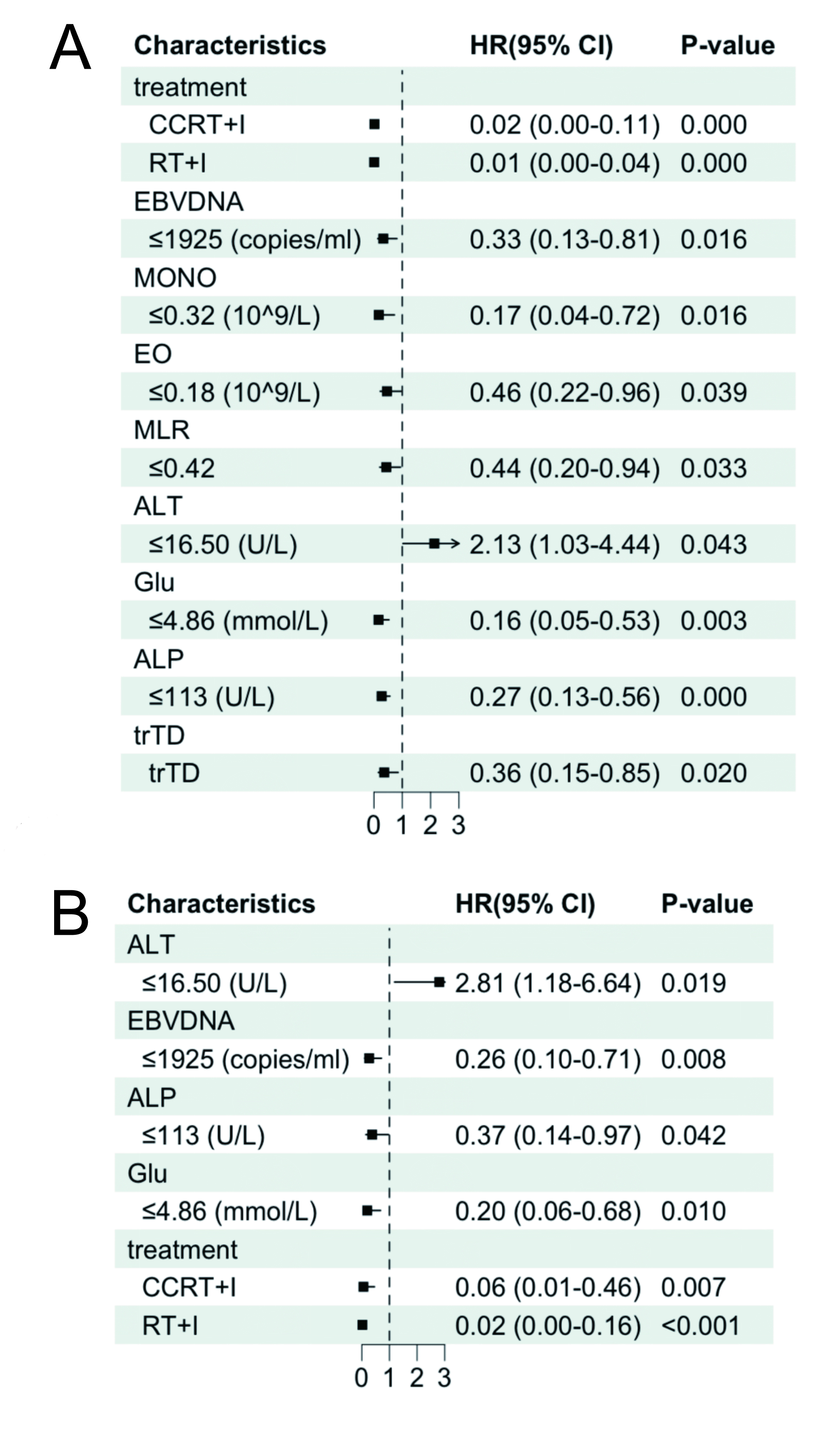

Supplement: oyag066_Supplementary_Data [file oyag066_supplementary_data.zip › Supplementary Figure3.tif]

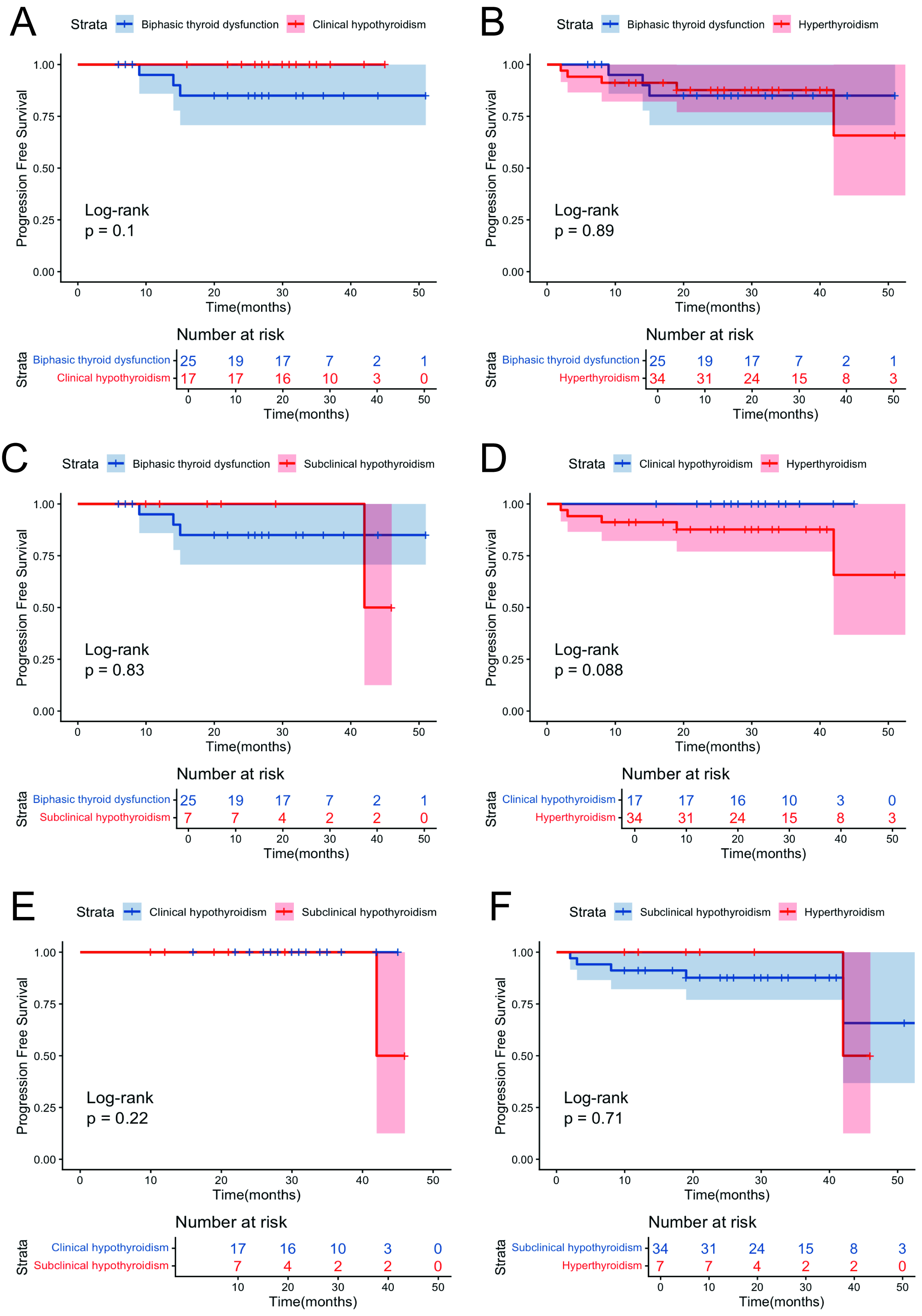

Supplement: oyag066_Supplementary_Data [file oyag066_supplementary_data.zip › Supplementary Figure_1.tif]

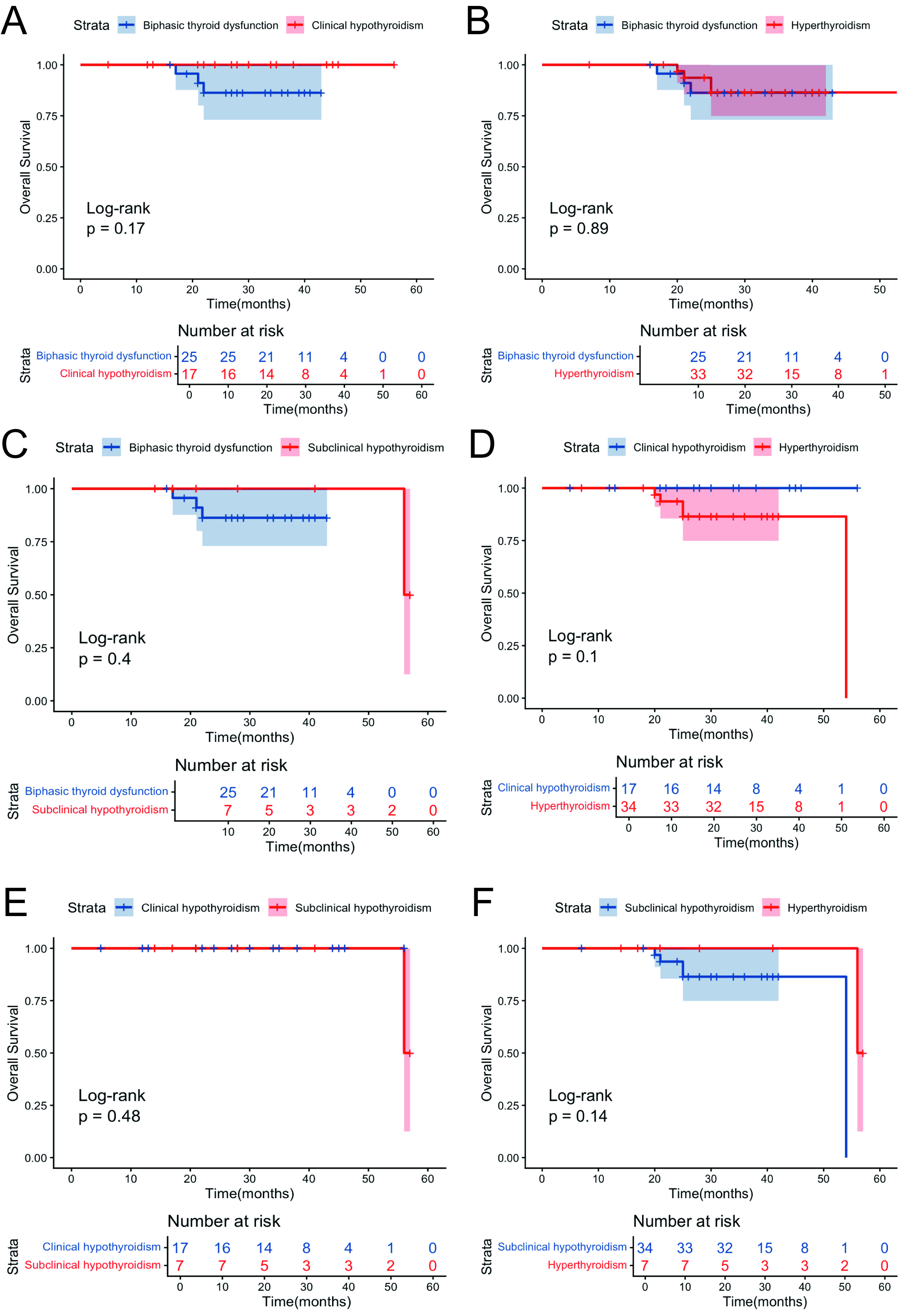

Supplement: oyag066_Supplementary_Data [file oyag066_supplementary_data.zip › Supplementary Figure_2.tif]
